# A prospective investigation of youth alcohol experimentation and reward responsivity in the ABCD study

**DOI:** 10.3389/fpsyt.2022.886848

**Published:** 2022-08-08

**Authors:** April C. May, Joanna Jacobus, Alan N. Simmons, Susan F. Tapert

**Affiliations:** ^1^San Diego Joint Doctoral Program in Clinical Psychology, San Diego State University/University of California, San Diego, San Diego, CA, United States; ^2^Department of Psychiatry, University of California, San Diego, San Diego, CA, United States

**Keywords:** alcohol experimentation, youth, nucleus accumbens, inferior frontal gyrus, machine learning, support vector machine, alcohol sipping

## Abstract

**Rationale:**

Greater risk-taking behaviors, such as alcohol experimentation, are associated with different patterns of brain functioning in regions implicated in reward (nucleus accumbens, NA) and cognitive control (inferior frontal gyrus, IFG). These neural features have been observed in youth with greater risk-taking tendencies prior to substance use initiation, suggesting NA-IFG disruption may serve as an early marker for subsequent substance use disorders. Prospective studies are needed to determine if NA-IFG neural disruption predicts future substance use in school-age children, including those with minimal use of alcohol (e.g., sipping). The present large-sample prospective study sought to use machine learning to: (1) examine alcohol sipping at ages 9, 10 as a potential behavioral indicator of concurrent underlying altered neural responsivity to reward, and (2) determine if alcohol sipping and NA-IFG activation at ages 9, 10 can be used to predict which youth reported increased alcohol use at ages 11, 12. Additionally, low-level alcohol use and brain functioning at ages 9, 10 were examined as predictors of substance use and brain functioning at ages 11, 12.

**Design and methods:**

This project used data from the baseline (Time 1) and two-year follow-up (Time 2) assessments of the Adolescent Brain Cognitive Development (ABCD) Study (Release 3.0). Support Vector Machine (SVM) learning determined if: (1) NA-IFG neural activity could correctly identify youth who reported alcohol sipping at Time 1 (*n* = 7409, mean age = 119.34 months, SD = 7.53; 50.27% female), and (2) NA-IFG and alcohol sipping frequency at Time 1 could correctly identify youth who reported drinking alcohol at Time 2 (*n* = 4000, mean age = 143.25 months, SD = 7.63; 47.53% female). Linear regression was also used to examine the relationship between alcohol sipping and NA-IFG activity at Time 1 and substance use and NA-IFG activity at Time 2. Data were also examined to characterize the environmental context in which youth first tried sips of alcohol (e.g., with or without parental permission, as part of a religious experience).

**Results:**

Approximately 24% of the sample reported having tried sips of alcohol by ages 9, 10. On average, youth reported trying sips of alcohol 4.87 times (SD = 23.19) with age of first sip occurring at 7.36 years old (SD = 1.91). The first SVM model classified youth according to alcohol sipping status at Time 1 no better than chance with an accuracy of 0.35 (balanced accuracy = 0.52, sensitivity = 0.24, specificity = 0.80). The second SVM model classified youth according to alcohol drinking status at Time 2 with an accuracy of 0.76 (balanced accuracy = 0.56, sensitivity = 0.21, specificity = 0.91). Linear regression demonstrated that frequency of alcohol sipping at Time 1 predicted frequency of alcohol use at Time 2 (*p* < 0.001, adjusted *R*^2^ = 0.075). Alcohol sipping at Time 1 was not linearly associated with NA or IFG activity at Time 2 (all *p*s > 0.05), and NA activity at Time 1 and Time 2 were not related (all *p*s > 0.05). Activity in the three subsections of the IFG at Time 1 predicted activity in those same regions at Time 2 (all *p*s < 0.02).

**Conclusions and implications:**

Early sips of alcohol appear to predict alcohol use in early adolescence. Findings do not provide strong evidence for minimal early alcohol use (sipping) as a behavioral marker of underlying alterations in NA-IFG neural responsivity to reward. Improving our understanding of the neural and behavioral factors that indicate a greater propensity for future substance use is crucial for identifying at-risk youth and potential targets for preventative efforts.

## Introduction

Approximately 10% of 8th graders in the United States report having used alcohol by the end of 6th grade (1). Over 40% of individuals who report drinking alcohol before age 14 become dependent on alcohol during their lifetime compared to 20% of those who initiate alcohol use after age 20 (2–5). Earlier substance use initiation is also linked to greater psychosocial difficulties (6–8), and increased risk for psychiatric disorders by young adulthood (9, 10). There is evidence to suggest a neural imbalance between cognitive control and motivational reward regions [e.g., nucleus accumbens (NA) and regions of the prefrontal cortex (PFC)] that is reflective of typical adolescent development (11). However, exposure to alcohol and drugs during adolescence does not always result in abuse or addiction. Individual differences in top-down regulation among some adolescents may increase the likelihood of engagement in risky behaviors and thus greater risk for negative outcomes (12). For example, among a sample of family-history positive youth, different neural patterns of brain response among cognitive control (i.e., dorsolateral PFC) and decision-making (i.e., posterior cingulate cortex) regions were found for youth classified as either resilient or high-risk based on substance use experiences by age 14 (13). Identification of early behavioral markers of underlying neural differences could be used to identify adolescents who may be particularly vulnerable to developing substance use problems prior to substance use initiation.

What is considered to be low-level alcohol use (< 4 drinks/month) among young adults (ages 19–21) has been found to effect response inhibition (14) but low-level alcohol use for youth between the ages of 9–12 has not been examined. Instead, research typically focuses on the role of age of substance use initiation and predictors of initiation in relation to future use and psychosocial problems. For example, a recent longitudinal study found that the odds of initiating alcohol use increase from ages 4 to 11 and that youth who are rated as behaviorally under controlled by their parents are more likely to initiate use (15). Further, earlier use is associated with an increased risk of binge drinking in high school (16). However, this literature has largely focused on youth who have consumed at least one full drink of alcohol, and studies vary as to whether youth who have only tried sips of alcohol are classified as abstainers or users (17). Understanding the impact of sipping alcohol at a very early age on development and future behaviors is important to determine whether very low-level alcohol use (i.e., sipping alcohol) can be considered an early indicator of vulnerability for future substance use problems.

Narrowly defining substance use initiation as the consumption of one full drink of alcohol has been identified as a short-coming that limits our ability to determine if sipping alcohol at a young age is prognostic of future risk-taking (17, 18). Studies demonstrate that youth who have a sip of alcohol at a young age (before age 10) are at greater odds of engaging in early onset drinking, defined as having one full drink or more (17, 18). To better understand this association between early sipping and increased risk of underage drinking, third grade children (mean age 9.2 years) and their parents completed a telephone interview. Youth answered questionnaires designed to assess attributes known to be associated with increased risk of underage drinking to determine if such differences can be observed at an early age. Those who reported higher self-esteem, behavioral self-regulation, and increased liking of school were found to be significantly less likely to report having already tried sips of alcohol while youth who had sipped alcohol were more likely to report breaking school rules and less likely to report self/identity satisfaction (19). Parental approval of drinking, parental drinking status, and children’s attitudes toward drinking have also been found to be predictive of sipping before age 10 (20). Despite differences in characterological attributes of youth who have not yet had a full drink of alcohol but engage in sipping, researchers have not fully investigated the neural differences between youth sippers and abstainers and how individual differences in reward-related brain responsivity may contribute to sipping behaviors.

Adolescence is a period of considerable neural development marked by cognitive and behavioral changes, including increased engagement in novelty-seeking and increases in risky decisions and risk-taking behaviors [e.g., substance use experimentation; (21–23)], suggesting that youth may weigh and experience rewards and risks differently than adults (24–28). Decisions are considered risky when they are made in uncertain situations without all relevant information and may potentially be associated with negative outcomes (29). Increased motivational drives to engage in risky behaviors can be seen as an adaptive response of becoming more independent and seeking out new experiences or environments. However, this behavioral change can also be maladaptive when it leads to experimentation and initiation of regular substance use (30).

While there are competing neural models, the imbalance model hypothesizes that this increase in risky behaviors is due to an imbalance in neurodevelopment among regions implicated in reward processing (striatum) and cognitive control [regions of PFC including inferior frontal gyrus (IFG); (11, 30)]. According to the imbalance model, PFC development increases linearly over time, contributing to limited cognitive control abilities during adolescence compared to adulthood. Alternatively, striatal regions (including the NA), implicated in detecting and learning about novel and rewarding environmental cues, follow a curvilinear pattern of development, with a peak in reward responsivity during adolescence. This exaggerated neural response in adolescents relative to adults can be seen in reward-processing brain regions [i.e., striatum, insula, anterior cingulate cortex; (31–34)] when anticipating and receiving various types of rewards including monetary (22) and pleasant tactile stimulation (35). Taken together, there is evidence for a weaker inhibitory control system coupled with a more developed striatal reward-processing system during adolescence. With age and neuromaturation, functional connectivity (defined as the temporal correlation in the signal between brain regions) increases and results in optimized top-down PFC (including IFG) monitoring of striatal (NA) regions. Behaviorally, developmental variations in NA-PFC imbalance may contribute to an increase in reward-seeking behaviors, including drug and alcohol experimentation (22) and increased susceptibility to the motivational properties of these substances.

Despite research identifying potential behavioral consequences of early alcohol sipping, very few studies have investigated whether there are neural differences between youth who do and do not sip alcohol. Differences in NA-PFC functional connectivity may differentiate those who sip alcohol at a young age from those who do not. Only one known study to date has examined risk-taking and reward-responsivity in youth who only tried a few sips of alcohol (36). Reward processing and risk-taking were studied using a monetary incentive delay (MID) task and the Cambridge Gamble Task (37), respectively, among 88 adolescents (mean age 14.5) with no lifetime substance use besides sipping alcohol on one or two occasions. Greater risk-taking performance on the Cambridge Gamble Task was associated with blunted neural activation in the ventral striatum (including NA) when anticipating a potential reward, suggesting that behavioral patterns of risk-taking are associated with altered neural patterns during reward for adolescents with very limited drinking history. As the study was cross-sectional, it was not determined whether such neural differences underlying risk-taking in a research setting are associated with real-life risk-taking behaviors over time (i.e., sipping alcohol, increased substance use). The present study is uniquely positioned to prospectively examine risk-taking within a research context in relation to the development of real-life risk-taking behaviors over time.

Machine learning (ML) utilizes existing information to detect patterns and build models that can be applied to future cases to accurately predict outcomes (38). Regression-based ML models can be used to predict continuous responses (e.g., amount of alcohol use) while Classification ML models predict group assignment (e.g., presence of a disorder such as depression). Classification models can be further categorized as supervised or unsupervised based on whether the training data used to build the model is labeled. In supervised ML, the model learns to classify data based on patterns identified in the labeled training dataset and then applies this learned grouping scheme to the test dataset. Alternatively, clustering represents an instance of unsupervised ML wherein data is grouped together based on similar features without any known information about group classification introduced in the training set. Two popular types of supervised classification ML models are Random Forests (RF) and Support Vector Machines (SVM). RF utilizes an ensemble of decision trees to classify data by building a model based on the majority vote across all individual trees (39). SVM is a robust prediction method that works to identify an optimal separation line, referred to as a hyperplane, between clusters of data points to identify groupings (40). These types of ML models have increasingly been applied in the field of substance use research. For example, Squeglia et al. (41) utilized RF classification models to identify demographic and behavioral features, such as gender, higher socioeconomic status, and positive alcohol expectancies, as most predictive of alcohol use by age 18. Similarly, Kinreich et al. (42) employed a Support Vector Machine (SVM) classifier to identify the most important biomarkers for predicting individuals most likely to develop an alcohol use disorder. In the research field of adolescent substance use, ML can be used to develop predictive models of negative substance use related outcomes (e.g., heavy use, development of use disorders) to aid in the early identification of youth at the greatest risk for future substance use problems.

The present large sample study seeks to utilize ML models to examine the predictive relationship between: (1) alcohol sipping at ages 9, 10 and underlying altered neural response to reward, and (2) alcohol sipping and neural activity at ages 9, 10 and alcohol use at ages 11, 12. Examining brain responsivity during reward processing as a function of individual differences in early sipping behavior is important for determining if adolescents who sip alcohol more frequently exhibit reward network differences that may leave them more vulnerable to future substance abuse. Additionally, low level alcohol use and brain functioning at ages 9, 10 (Time 1) will be examined as a predictor of future substance use and altered brain functioning at two-year follow-up (Time 2; ages 11, 12) in regions implicated in reward (NA) and cognitive control (IFG). Four specific hypotheses will be tested: (1) Time 1 NA-IFG activation during a MID reward task can be used to accurately classify youth who report having sipped alcohol at Time 1 (ages 9, 10); (2) Greater sipping frequency at Time 1 (ages 9, 10) will be linked to greater substance use at Time 2 (ages 11, 12); (3) MID-elicited NA-IFG functioning during reward trials and sipping frequency at Time 1 (ages 9, 10) can be used to accurately classify youth drinking status at Time 2 (ages 11, 12); and (4) Greater sipping frequency and high NA-low IFG activity during reward at Time 1 (ages 9, 10) will prospectively predict high NA-low IFG MID-elicited activity during reward trials at Time 2 (ages 11, 12).

Overall, this project seeks to determine if early but minimal alcohol exposure to alcohol is reflective of underlying differences in brain functioning and predictive of future substance use. Testing these hypotheses will contribute to our understanding of the progression of substance use in youth.

## Materials and methods

This study utilized data gathered as part of the Adolescent Brain and Cognitive Development (ABCD) consortium, the largest national, multi-site, longitudinal study to date aimed at prospectively examining brain and cognitive development among youth. A nationally representative sample of 11,878 9- and 10-year-old children were enrolled between September 2016 and August 2018 at 21 sites across the country. ABCD employed a rigorous epidemiological recruitment approach to ensure diversity across the sample (43). Within the catchment area for each of the 21 sites, individual schools and school districts that match demographic targets based on sociodemographics (sex, race, ethnicity, urbanicity, and socioeconomic status) were selected as recruitment targets. To specifically examine the effects of substance use on adolescent development, the sample was enriched with youth considered to be at high risk for future substance use disorder based on three characteristics: (1) high rates of externalizing symptoms; (2) smoking in the home; and (3) endorsement of negative affect (44). This oversampling procedure was employed to help ensure the presence of substance use initiation in the sample. The cohort will be actively followed for 10 years with youth completing annual in-person follow-up assessments as well as annual phone-based follow-ups starting at 6 months post-baseline. Thus far, participants have been followed up with at a rate of 98%.

### Participants

Youth were between the ages of 8.9 to 11.1 years of age at study enrollment, required to be fluent in English, and able to validly and safely complete all study assessments including magnetic resonance imaging (MRI) scanning. Baseline youth exclusion criteria for the overall ABCD consortium included: (*a*) no biological parent or legal guardian to provide permission; (*b*) a current diagnosis of schizophrenia, autism spectrum disorder (moderate, severe), intellectual disability, or alcohol/substance use disorder; (*c*) parental indication that child is not be capable of following instructions and completing the protocol; (*d*) non-correctable vision, hearing, or sensorimotor deficits; (*e*) presence of a major medical condition (e.g., cerebral palsy, brain tumor, stroke); (*f*) Gestational age less than 28 weeks, and birth weight less than 1.2 kilograms (2 lbs. 10 oz.); (*g*) birth complications that resulted in being hospitalized for more than one month; (*h*) a history of traumatic brain injury with loss of consciousness > 30 min, amnesia > 24 h, or positive neuroimaging findings; and (*i*) MRI contraindications including irremovable ferromagnetic dental appliances on other mental implants, claustrophobia, or pregnancy (43).

### Preliminary analyses

Based on a preliminary analysis of the full baseline sample (*N* = 11878), approximately 24% reported having tried at least one sip of alcohol. On average, youth reported having 4.7 sips (*M* = 4.7, SD = 19.2, median = 1), and 1.6% reported finishing the drink after having the first sip. The majority of youth reported taking a sip of beer (41%) or wine (30%) that belonged to their mother (37%) or father (42%). These preliminary analyzes demonstrate that a sufficient number of youths enrolled in ABCD reported having tried sips of alcohol at baseline to complete the proposed analyzes.

### Procedures

At Time 1, all youth completed a thorough assessment session including a clinical interview and other measures administered by a trained research assistant (RA) to gather information related to psychopathology, substance use, family history, and general background information (Table 1). Youth also completed an MRI scan session during which anatomical and functional series were acquired. Similar mental health, family history, and general background information was gathered from a biological parent or legal guardian by a separate RA. The two-year follow-up assessment (Time 2; ages 11, 12) follows a similar structure to that of baseline. If any MRI contraindications prevents MRI completion (e.g., braces), staff members work with families to delay MRI if possible and/or collect other elements of the protocol. If a family moves away, efforts are made to schedule their follow-up assessments at a more convenient ABCD site.

**TABLE 1:** Timeline of measures administered and domains assessed (65).

ABCD assessments	What it measures	Baseline	2-YR FU (Time 2)	Who
Demographics survey (65)	Demographics, race, gender, family structure, SES, education	X	X	P
Timeline followback (49)	Quantity/frequency of all substance use	X	X	Y
iSay II Q2 sipping items (18)	Early alcohol use	X	X	Y
Monetary incentive delay functional MRI task (66, 67)	Neural processing of reward	X	X	Y

2-YR FU, two-year follow-up assessment/time 2; P, parent; Y, youth.

#### Scan sessions

Scan sessions are conducted at each ABCD site using either a GE MR750, Siemens PRISMA or Philips scanner, all equipped with a 32-channel head coil. See Table 2 for scan parameters. A 3D T1-weighted magnetization-prepared rapid acquisition gradient-echo scan using prospective motion correction (PROMO) is used to obtain a high-resolution anatomical image for cortical and subcortical segmentation. PROMO technology allows for ability to correct for motion in real-time (45) and has been demonstrated as particularly beneficial for scanning children who have more difficulty staying still (46, 47). For further review of imaging acquisition and processing methods used in the ABCD study see Hagler et al. (48).

**TABLE 2:** Scan parameters for each type of scanner used across the 21 sites (53, 48).

	Matrix	Slices	FOV	Resolution (mm)	TR (ms)	TE (ms)	TI (ms)	Flip Angle (deg)	Total Acq. Time
GE T1	256 × 256	208	256 × 256	1.0 × 1.0 × 1.0	2500	2	1060	8	6:09
Siemens T1	256 × 256	176	256 × 256	1.0 × 1.0 × 1.0	2500	2.88	1060	8	7:12
Philips T1	256 × 256	225	256 × 240	1.0 × 1.0 × 1.0	6.31	2.9	1060	8	5:38
2 fMRI runs for MID task (across platforms)	90 × 90	60	216 × 216	2.4 × 2.4 × 2.4	800	30.0	N/A	52	11:10

FOV, field of view; TR, repetition time; TE, echo time; TI, inversion time.

### Measures

#### Demographics

The Modified PhenX Demographics Survey was administered to all participants’ parents or caregivers at baseline. This measure captures basic demographic information including race, ethnicity, and gender. Further information was collected pertaining to the youths’ family structure, socioeconomic status, and education level of the child and family members.

#### Substance use

The iSay II Q2 Sipping Items (18) was administered at baseline for youth who endorsed having had at least one sip of alcohol. Youth provided information regarding: (1) total number of times they have had a sip of alcohol; (2) whether and how many times they have had sips of alcohol as part of a religious ceremony; and (3) age at first sip of alcohol. If the youth did not report sipping alcohol at baseline, this measure was assessed again at Time 2. Of note, the current study examined all alcohol sipping regardless of context (i.e., as part of a religious ceremony or not) as previous research has suggested that any experimentation with alcohol may set a trajectory for future use if drinking is perceived to be socially acceptable (17). Once youth begin to engage in substance use, The Drug Intro and Timeline Followback (49) is administered to gather information about use of alcohol and other substances including: (1) age of first use and regular use; (2) lifetime quantity; (3) maximum use in one sitting; and (4) last use. At follow-up, for youth with lifetime use of any substance 6 + times, additional measures will characterize use patterns. The Timeline Followback (49) uses a calendar-based capture of substance use, including age of first use, frequency (per month), quantity, modes, specific products/potency, social context, and source of substances. To facilitate accurate labeling and quantification, pictures (e.g., shatter, dabs/wax, oil, hash, and flower for cannabis; standard drink sizes for alcohol) are presented.

#### Neural processing of reward

**FIGURE 1:**
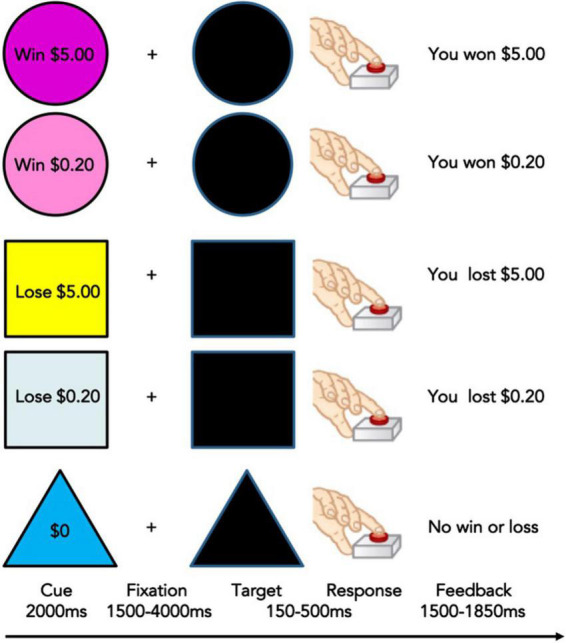
Monetary Incentive Delay Task used in the Adolescent Brain and Cognitive Development (ABCD) Study (53). Adapted from Knutson et al. (50).

A Monetary Incentive Delay (MID) task is collected during functional MRI at Time 1 (baseline) and Time 2 (2 year follow-up). This task measures reward processing and motivation including anticipation and receipt (or loss) of reward and requires speeded responses to win or avoid loss (50). It shows developmental and addiction-related effects and possesses reliability over time. The MID task has also been shown to reliably activate well-characterized regions of the reward network including ventral striatum and NA, and cognitive control regions including ACC, PFC, and IFG (50). Various MID task versions have also been used with adolescents, demonstrating its developmental appropriateness (51, 52). Therefore, this task is a well-validated functional task to elicit neural responses to reward stimuli in youth. Subjects see incentive cues associated with four possible gains or losses ($0.20, $5, -$0.20, -$5, $0), followed by an anticipation delay, then a target period during which subjects must press a button to gain or avoid losing money (Figure 1). If participants press too quickly or too slowly, they will lose the previously indicated amount of money. Feedback is given on each trial and the task is administered over two runs. For additional details of the scan sessions and MID task in particular please see Cho et al. (53).

### Image processing

**FIGURE 2:**
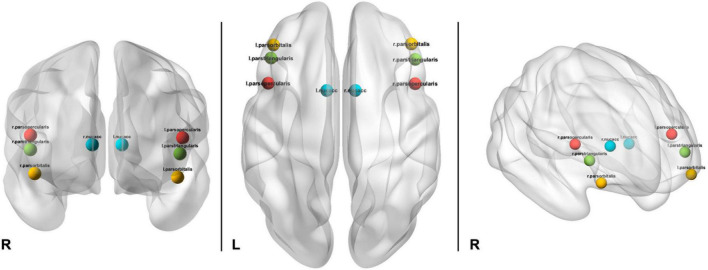
Depiction of location of ROIs used in analyzes including bilateral pars opercularis (red), pars triangularis (green), pars orbitalis (yellow), and nucleus accumbens (aqua).

Imaging data processing, including motion correction, was conducted by the ABCD Data Analytics and Informatics Resource Center (DAIRC), based at UC San Diego (48). All processed data and tabulated region of interest-based analysis results are publicly available via the National Institute for Mental Health Data Archive (NDA). For further details of all neuroimaging processing and subject-level analysis methods used by ABCD please see Hagler et al. (48). Briefly, preprocessing includes: (1) Estimation of B_0_ distortion field from spin echo images and applied to each gradient echo frame after accounting for head motion; (2) Correction of Functional MRI data using standard volume-wise methods; (3) Registration between T_2_*-weighted fMRI images and T_1_-weighted structural images using mutual information; (4) Slice-time correction and volume registration. Cortical regions of interest were defined according to the Desikan atlas (54) and subcortical regions were labeled using atlas-based segmentation (55). The present analyzes included the three bilateral cortical segments comprising the inferior frontal gyrus, pars opercularis (PP), pars triangularis (PT), and the pars orbitalis (PB), as well as the bilateral NA (Figure 2).

A general linear model was conducted to estimate MID task-related activation using AFNI’s 3dDeconvolve. The MID model included predictors for anticipation of large, small, and no reward and feedback for large, small, and no reward for wins and losses. The linear contrast of positive (i.e., large or small reward) versus negative feedback (i.e., failure to win small or large reward) was used for the present analyzes.

### Data analysis

#### Data preparation

**FIGURE 3:**
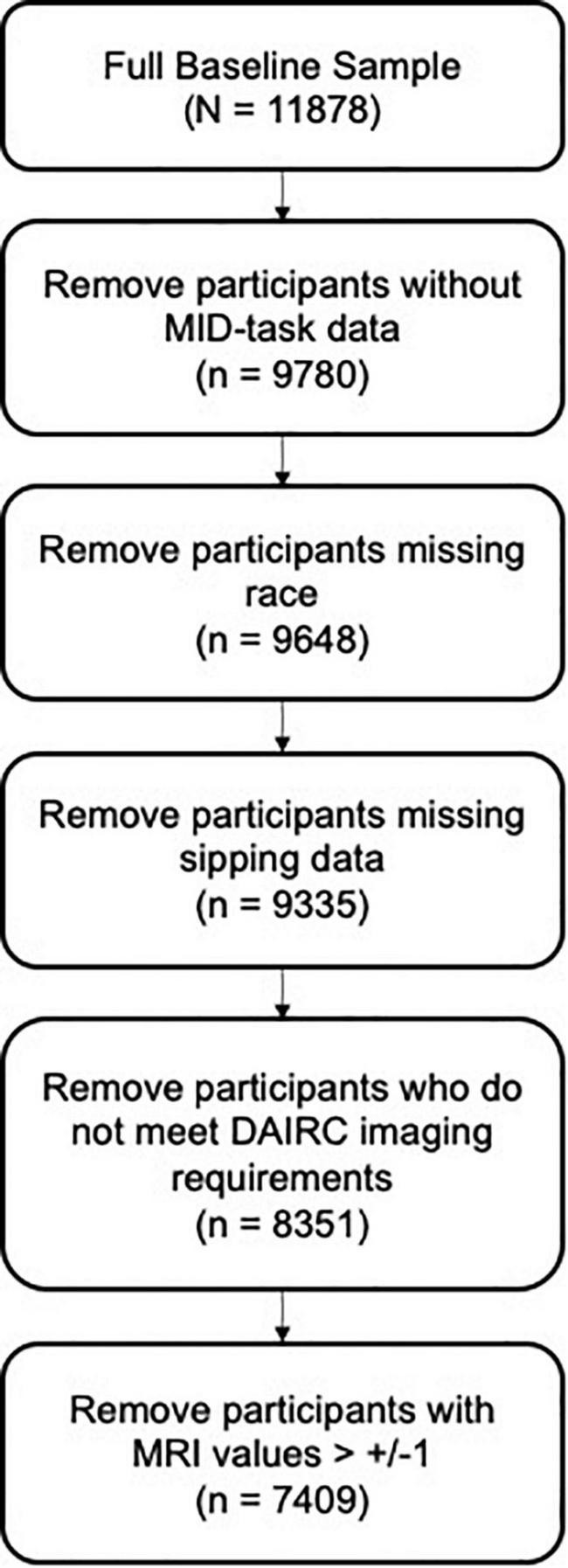
Data cleaning steps for Hypothesis 1 resulting in a subsample of *n* = 7409.

All data analyzes were conducted in R Statistical Program using ABCD Data Release 3.0. Data on reported race were recoded into one variable with four response levels: white, black, asian, and other/mixed. The total sample was pared down to only include participants who completed the MID-task, the iSay II Q2 Sipping Items, and reported race. ABCD DAIC recommendations for MRI task-based quality control were followed and participants who did not meet DAIC requirements were excluded from the present analyzes (see Figure 3). MRI data was further cleaned to remove any participants with outlier values (outside the range of ± 1).

The ComBat function of the ‘sva’ R package was employed to remove any batch effects due to different scanners and to account for differences in age and gender between sites using a parametric empirical Bayesian framework. To remove the effects of age, gender, and age^2^ from the feature set, the residuals of each were calculated and then removed. Additionally, a variable was created to indicate Alcohol Drinking Status at Time 2 to be used in Hypothesis 3. Any past year use of alcohol greater than zero was recoded as a ‘1’ to indicate the youth reported some form of alcohol use in the past year.

#### Machine learning models (Hypotheses 1 & 3)

**FIGURE 4:**
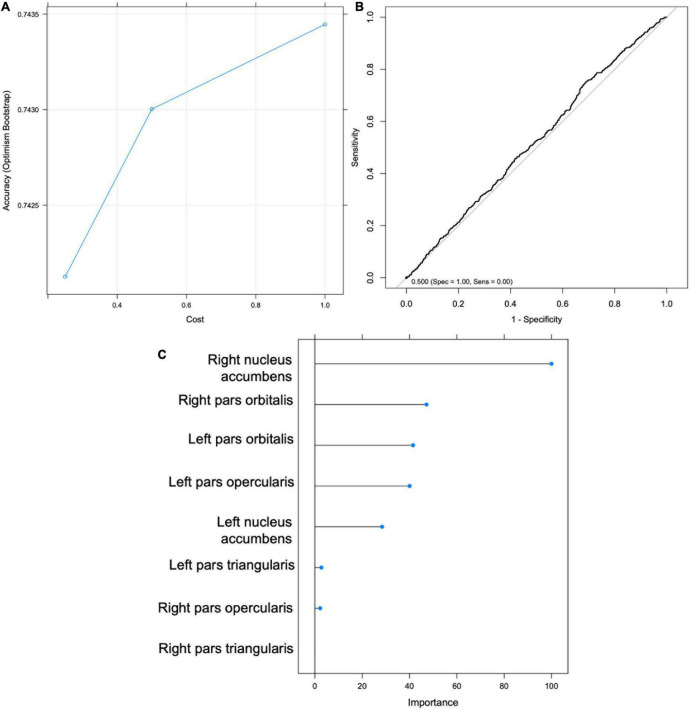
Support Vector Machine (SVM) results for Hypothesis 1. **(A)** Graph depicting accuracy vs. cost for model selection. **(B)** Receiver Operating Characteristic (ROC) Curve. **(C)** Importance scores for each feature.

Support Vector Machine models were trained to test Hypotheses 1 and 3. All machine learning models were run using the R ‘caret’ package using the Support Vector Machine with Radial Basis Function Kernel (‘svmRadial’) method. Analytical decisions were based on standard procedures and recommendations outlined in Kuhn and Johnson (38). Data were partitioned to create a stratified random sample of the full dataset into training (67% of the dataset) and test (33% of the dataset) sets based on the outcome variable of interest (e.g., sipping or alcohol status) to ensure balanced splits of the data in each subsample. Preprocessing transformations (i.e., centering, scaling) were estimated from the training sample using the caret package (‘preProcess’) and then applied to the test sample. The model was further tuned using the ‘trainControl’ function of the caret package which utilizes a bootstrap estimator (‘optimism_boost’). For the bootstrap estimate, a random 75% of the training set was selected on which the classified was trained (i.e., 75% of the 67% of the full sample, or *n* = 3719) and then tested on the remaining 25% of the training set; this process was repeated 20 times on random 75/25 splits. To evaluate the model Receiver Operating Characteristic (ROC) curves were plotted (see Figure 4) presenting the sensitivity (true positives) and specificity (true negatives) at various thresholds of the binary classifier. Classification accuracy, the ratio of sum of true positives and true negatives divided by the sum total of all classified participants, was the chosen metric for selecting the optimal model. Balanced accuracy was also reported which takes the unbalanced proportion of sippers to non-sippers into account and adjusts accordingly.

For hypothesis 1, baseline activation in bilateral inferior frontal gyrus (parcellated into 3 regions) and bilateral NA were included as the 8 features by which to classify youth according to sipping status at Time 1. Hypothesis 3 included the same 8 baseline MRI variables as well as baseline sipping status as features to classify youth based on drinking status at Time 2.

#### Regression models (Hypotheses 2 & 4)

For Hypothesis 2, a set of three separate regressions with alcohol, cannabis, and nicotine use at Time 2 (reported as a continuous variable of frequency of use) as the DV for each regression. Sipping frequency at baseline was the predictor variable of interest.

For Hypothesis 4, a series of 8 multiple regressions were run with a single DV of the region of interest (i.e., right or left NA or the 3 regions of the IFG). Sipping frequency at Time 1 and neural activity in the bilateral NA and bilateral IFG regions at Time 1 were the IVs for each regression.

## Results

Data from a total of 7,409 youth (50.27% female) with an average age of 119.34 months (SD = 7.53; Table 3) were used to test Hypothesis 1. Approximately 24% of the sample reported having tried sips of alcohol by ages 9, 10. Of those who reported trying sips of alcohol, 76% reported doing so outside of the context of a religious ceremony. As this is the first known study to examine the relationship between neural responsivity and low-level alcohol use, analyzes examined all alcohol sipping regardless of context. On average, youth reported trying sips of alcohol 4.87 times (SD = 23.19) with age of first sip occurring at an average age of 7.36 years old (SD = 1.91). Given the timing of the ABCD Data Release 3.0, only 4000 youth were used to test Hypotheses 2–4. These hypotheses required data from the Time 2 assessment which had not been completed by the entire sample in time for public release 3.0. The average age of the participants at Time 2 was 143.25 months (SD = 7.63) and the sample was 47.53% female (see Table 3).

**TABLE 3:** Participant demographics and substance use history characteristics.

Participants at Time 1 (*N* = 7409)	*M* (SD)/%	Range
Age at Time 1 (months)	9.95 (0.63)	8.92–11.08
% Female	50.27%	–
White	68.98%	–
Black	15.18%	–
Asian	6.13%	–
Other/Mixed	9.71%	–
**Participants at Time 2 (*N* = 4000)**	***M* (SD) or%**	**Range**

Age at Time 2 (years)	11.94 (0.64)	10.6–13.58
% Female	47.53%	–
**Substance use at Time 1 (*n* = 7409)**	***M* (SD)/%**	**Range**

% Endorsing any alcohol sipping	23.87%	–
Alcohol sipping frequency	4.44 (14.18)	1–260
**Substance use at Time 2 (*n* = 4000)**	***M* (SD)/%**	**Range**

% Reporting alcohol use in past year	10.45%	–
Alcohol sipping in past year	0.58 (4.28)	0–104
Cannabis use in past year	0.01 (0.44)	0–25
Nicotine use in past year	0.03 (0.73)	0–35

### Hypothesis 1

#### Support vector machine (SVM) classification accuracy and top significant variables

An SVM was trained on 67% of the total sample (*n* = 4959). The optimal model was selected to maximize classification accuracy at 0.742 with tuning parameters set at cost = 1 and sigma = 0.155, yielding a kappa of -0.077 (Figure 4). The model was then validated on the test set (*n* = 2442). Using the mean probability value of 0.24, the model had an accuracy of 0.35 (95% CI: 0.335 -0.373), balanced accuracy of 0.52, sensitivity of 0.244, and specificity of 0.795 (Figure 4). The right NA was selected as the most important predictor variable (Figure 4). The ROC curve (Figure 4) indicates that the model was approximately equal to chance at classifying youth’s Time 1 sipping status; therefore Hypothesis 1 was not supported. Additionally, an exploratory follow-up analysis was conducted wherein an SVM was trained only on youth who reported sipping alcohol outside of a religious context. Findings were similar with the model performing no better than chance at correctly classifying youth’s Time 1 sipping status (balanced accuracy of 0.410).

### Hypothesis 2

#### Alcohol use

At Time 2, the mean amount of alcohol use in the past year was 0.58 (SD = 4.28) with the modal value reported being zero (Table 3). A total of 418 youth reported using alcohol in the past year at Time 2. A linear regression demonstrated that frequency of alcohol sipping at Time 1 significantly predicted frequency of alcohol use at Time 2, *F* (1,3998) = 321.6, *p* < 0.001, with sipping at Time 1 explaining 7.5% of the variability in alcohol use at Time 2 (Table 4). Given the inclusion of youth who sip alcohol within a religious context, an additional exploratory regression was run only within the subset of the sample who predicted sipping alcohol outside of a religious context and had completed the Time 2 follow-up, to confirm that these findings were not solely driven by youth sipping in such contexts. This follow-up linear regression produced similar findings, suggesting that frequency of alcohol sipping at Time 1 significantly predicted frequency of alcohol use at Time 2 among youth who reported alcohol sipping *only* outside of a religious context [*F* (1,3926) = 192.7, *p* < 0.001, *R*^2^ = 0.047].

**TABLE 4:** Regression results for Hypothesis 2 demonstrating a significant predictive relationship between frequency of alcohol sipping at Time 1 and frequency of alcohol use, but not cocaine or nicotine use (due to insufficient use levels), at Time 2.

	Alcohol (*B*, SE)	Cannabis (*B*, SE)	Nicotine (*B*, SE)
Intercept	0.578 (0.065)***	-0.00000672 (0.00698)	0.0000105 (0.0116)
Alcohol Sipping Freq.	0.073 (0.004)***	-0.000059 (0.000435)	-0.0000870 (0.000723)
*F*	321.6***	0.018	0.015
DF	1, 3998	1, 3998	1, 3998
*R* ^2^	0.074	0.00000461	0.00000363
Adjusted *R*^2^	0.074	-0.0002455	-0.0002465

***p < 0.001.

#### Cannabis use

Only 14 youth reported cannabis use in the past year at Time 2 with a mean of 0.01 (SD = 0.44; see Table 3). A regression was run on the full dataset which did not find a significant relationship between early sipping and future cannabis use, *F* (1,3998) = 0.018, *p* = 0.892 (Table 4). Given the minimal amount of cannabis use in the sample at Time 2, this data cannot adequately address the hypothesis.

#### Nicotine use

Nicotine use in the past year was reported by 19 youth at Time 2 (*M* = 0.03, *SD* = 0.73; Table 3). Of the 19 youth who reported nicotine use at Time 2, 9 reported sipping alcohol at Time 1 (number of sips ranged from 1 to 4). Again, the minimal past year nicotine use reported by youth limits the ability to adequately address the hypothesis. However, no significant relationship was found between the alcohol sipping at Time 1 and nicotine use at Time 2, *F* (1, 3998) = 0.015, *p* = 0.91 (see Table 4).

### Hypothesis 3

#### Support vector machine (SVM) classification accuracy and top significant variables

**FIGURE 5:**
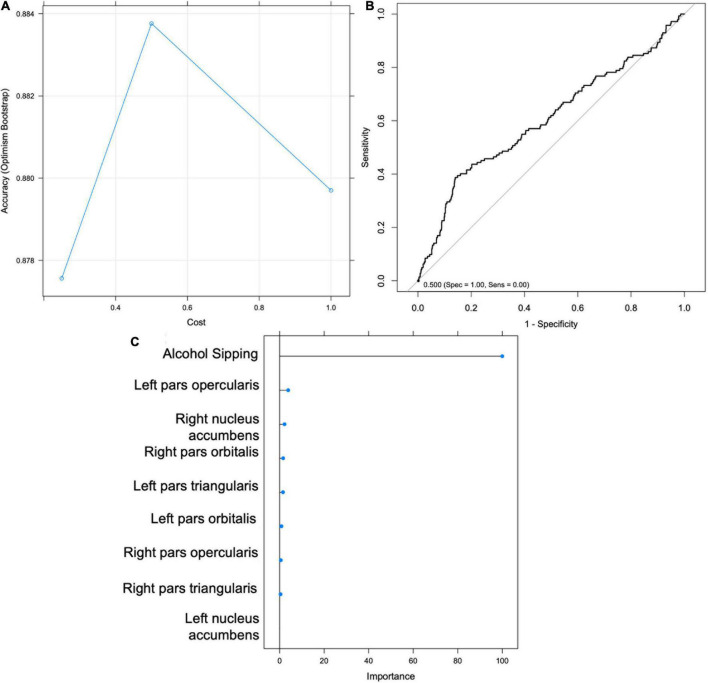
Support Vector Machine (SVM) results for Hypothesis 3. **(A)** Graph depicting accuracy vs. cost for model selection. **(B)** Receiver Operating Characteristic (ROC) Curve. **(C)** Importance scores for each feature.

An SVM model was trained to classify youth according to drinking status at Time 2 using MRI variables (IFG and NA) and sipping frequency at Time 1. The optimal model yielded a classification accuracy of.886 and kappa of -0.038, with tuning parameters set at cost = 0.25 and sigma = 0.124 (Figure 5). The model was then validated on the test set (*n* = 1321) and yielded an accuracy of.758 (95% CI: 0.734–0.781) and balanced accuracy of 0.56, when using a median probability of 0.11. Again, the results indicate that the model is no better than chance at accurately classifying youth drinking status at Time 2 and therefore do not support Hypothesis 3. The model accurately classified 21% of youth drinkers (sensitivity) and 91% of non-drinkers (specificity). Sipping frequency at baseline was identified as the most important classification variable (Figure 5). Without sacrificing specificity, the model achieved a maximum sensitivity of approximately 0.42 which corresponds with a specificity of approximately 0.80 (Figure 5).

### Hypothesis 4

#### Nucleus accumbens activity at Time 2

A multiple regression found no significant overall relationship between alcohol sipping, NA neural activity, and IFG neural activity at Time 1 and right NA [*F* (9, 2832) = 1.21, *p* = 0.28] or left NA [*F* (9, 2832) = 1.49, *p* = 0.15] activity at Time 2 (Table 5). Although the overall model was not significant, sipping at Time 1 was found to be a significant predictor (*p* = 0.005) of neural activity in the left NA at Time 2. Therefore, the model was respecified to examine the direct relationship between alcohol sipping at Time 1 and left NA activity at Time 2. The follow-up analysis revealed frequency of alcohol sipping at Time 1 to significantly predict neural activity in the left NA at Time 2, *F* (1,2840) = 7.81, *p* = 0.005, however, alcohol sipping at Time 1 only explained.27% of the variability in left NA activity at Time 2.

**TABLE 5:** Multiple regression results for Hypothesis 4 predicting linear relationships between alcohol sipping frequency and bilateral nucleus accumbens (NA) and inferior frontal gyrus (IFG) activity at Time 1 and bilateral NA and IFG activity at Time 2.

	Right NA (*B*, SE)	Left NA (*B*, SE)	Right PP (*B*, SE)	Left PP (*B*, SE)
Intercept	0.004 (0.005)	0.005 (0.005)	-0.0003 (0.004)	0.0003 (0.003)
Alcohol Sipping Freq.	-0.0001 (0.0002)	-0.001 (0.001)**	0.0001 (0.0002)	-0.00006 (0.0002)
Right NA at Time 1	-0.010 (0.026)	-0.012 (0.026)	-0.027 (0.019)	-0.039 (0.019)*
Left NA at Time 1	0.043 (0.025)	0.041 (0.025)	0.007 (0.019)	0.009 (0.018)
Right PP at Time 1	0.055 (0.047)	0.021 (0.047)	0.015 (0.035)***	0.012 (0.034)
Left PP at Time 1	-0.038 (0.052)	0.033 (0.052)	0.049 (0.039)	0.019 (0.037)***
Right PB at Time 1	0.016 (0.021)	-0.0004 (0.021)	0.014 (0.016)	0.008 (0.015)
Left PB at Time 1	0.027 (0.022)	0.011 (0.022)	-0.009 (0.016)	-0.007 (0.016)
Right PT at Time 1	-0.021 (0.039)	-0.031 (0.039)	-0.047 (0.029)	-0.043 (0.028)
Left PT at Time 1	-0.045 (0.038)	-0.021 (0.038)	-0.056 (0.029)	-0.048 (0.028)
*F*	1.21	1.49	4.98***	5.99***
DF	9,2832	9,2832	9,2832	9,2832
*R* ^2^	0.004	0.005	0.016	0.019
Adjusted *R*^2^	0.001	0.002	0.012	0.016
	**Right PB** **(*B*, SE)**	**Left PB** **(*B*, SE)**	**Right PT** **(*B*, SE)**	**Left PT** **(*B*, SE)**
Intercept	0.0003 (0.005)	0.001 (0.005)	-0.002 (0.004)	-0.0002 (0.004)
Alcohol Sipping Freq.	0.0001 (0.001)	-0.001 (0.001)	0.0003 (0.0002)	-0.0001 (0.0002)
Right NA at Time 1	-0.009 (0.029)	-0.003 (0.029)	-0.016 (0.022)	-0.004 (0.022)*
Left NA at Time 1	-0.022 (0.028)	-0.023 (0.028)	0.003 (0.021)	-0.008 (0.021)
Right PP at Time 1	0.055 (0.053)	-0.017 (0.053)	0.003 (0.039)	0.008 (0.040)
Left PP at Time 1	0.007 (0.059)	0.084 (0.058)	0.035 (0.043)	0.027 (0.045)
Right PB at Time 1	0.120 (0.024)***	0.0008 (0.024)	0.019 (0.018)	0.015 (0.018)
Left PB at Time 1	-0.035 (0.025)	0.081 (0.024)***	-0.015 (0.018)	-0.015 (0.019)
Right PT at Time 1	-0.079 (0.045)	-0.040 (0.044)	0.076 (0.033)*	-0.021 (0.034)
Left PT at Time 1	0.018 (0.044)	-0.024 (0.043)	-0.016 (0.032)	0.112 (0.033)***
*F*	3.49***	2.61***	3.31***	4.59***
DF	9,2832	9,2832	9,2832	9,2832
*R* ^2^	0.011	0.008	0.010	0.014
Adjusted *R*^2^	0.008	0.005	0.007	0.011

NA, nucleus accumbens; PP, pars opercularis; PB, pars orbitalis; PT, pars triangularis. SE, standard error; DF, degrees of freedom. *p < 0.05, **p < 0.01, ***p < 0.001.

#### Inferior frontal gyrus activity at Time 2

##### Pars opercularis

Two separate multiple regressions were run to predict neural activity in the left and right PP at Time 2 from alcohol sipping, bilateral NA activity, and bilateral IFG activity at Time 1. The first multiple regression model significantly predicted activity in the left PP [*F* (9, 2832] = 5.99, *p* < 0.001, adj. *R*^2^ = 0.02; Table 5). Neural activity in the right NA (*B* = −0.038, *p* = 0.037) and left PP (B = 0.193, *p* < 0.001) at Time 1 were both found to significantly contribute to the model while the other predictors did not. The second multiple regression model demonstrated a significant relationship between the right PP and the predictors listed above [*F* (9, 2832) = 4.98, *p* < 0.001, adj. *R*^2^ = 0.01], however, the only significant individual predictor was right PP activity at Time 1 (*B* = 0.15, *p* < 0.001).

##### Pars orbitalis

Two separate multiple regression models were run to predict left and right PB. The first multiple regression demonstrated a significant relationship between NA/IFG activity and alcohol sipping at Time 1 with left PB activity at Time 2 [*F* (9, 2832) = 2.61, *p* = 0.005, adj. *R*^2^ = 0.005; Table 5]. However, the only significant individual predictor was left PB activity at Time 1 (*B* = 0.08, *p* < 0.001). A second regression demonstrated a significant relationship between the predictor variables and right PB activity at Time 2 [*F* (9, 2832) = 3.49, *p* < 0.001, adj. *R*^2^ = 0.007]. Similarly, right PB activity at Time 1 was the only significant predictor (*B* = 0.12, *p* < 0.001).

##### Pars triangularis

A multiple regression model demonstrated a significant relationship between NA/IFG activity and alcohol sipping at Time 1 with left PT activity at Time 2 [*F* (9, 2832) = 4.59, *p* < 0.001, adj. *R*^2^ = 0.01; Table 5]. Right NA activity (*B* = −0.04, *p* = 0.04) and left PT activity (*B* = 0.11 *p* < 0.001) at Time 1 were the only significant individual predictors. Similarly, a multiple regression demonstrated an overall significant relationship between the predictor variables and PT activity at Time 2 [*F* (9, 2832) = 3.31, *p* < 0.001, adj. *R*^2^ = 0.007]. Right PT activity at Time 1 was the only significant individual predictor (*B* = 0.08 *p* = 0.019).

## Discussion

Utilizing data from the ABCD study, this project aimed to prospectively examine if early but minimal alcohol use (i.e., taking sips at ages 9, 10) is associated with an altered neural response to reward in the NA and IFG and predictive of higher levels of substance use in the future. Specifically, it was hypothesized that: (1) NA-IFG activation during a MID reward task at Time 1 could predict youth sipping status; (2) greater sipping frequency at Time 1 would be associated with greater substance use (alcohol, cannabis, nicotine) at Time 2; (3) MID-elicited NA-IFG activity and sipping frequency at Time 1 could predict youth drinking status at Time 2; and (4) greater sipping frequency and high NA-low IFG activity during reward at Time 1 would be associated with high NA-low IFG at Time 2 (ages 11-12). These hypotheses were partially supported as follows.

Using machine learning, Hypothesis 1 was tested to see if NA and IFG activity in response to positive versus negative feedback on reward trials could classify youth on whether or not they reported having tried sips of alcohol. Support for this hypothesis would suggest that early alcohol sipping is a behavioral indicator of underlying features in neural responsivity to reward. The SVM model identified the right NA as the most important feature for classifying youth based on sipping status, consistent with the literature demonstrating an association between NA activation and greater risk-taking and alcohol consumption (36, 56). However, the model correctly identified only 24% of youth sippers when applied to the test data set, suggesting a lack of strong evidence for an alcohol sipping and reward activation association. Early alcohol sipping may instead be more influenced by outside environmental factors (e.g., parental use, parental attitudes about alcohol use, or perceived peer norms) rather than variations in neural responsivity.

Hypothesis 2 proposed that alcohol sipping at ages 9, 10 (Time 1) would predict alcohol, cannabis, and nicotine use at Time 2. The number of youth reporting cannabis (*n* = 14) and nicotine (*n* = 19) use at Time 2 were insufficient for conducting the proposed analyzes. Although youth are still relatively young at Time 2 (ages 11, 12) higher rates of cannabis and nicotine use were anticipated by this age given previous findings. For example, Rioux et al. (57) found 4% of their sample had used cannabis by age 13 while Malmberg et al. (58) examined a sample of over 3000 youth and found average age of onset for cannabis to be 12.45 (SD = 0.74) and 11.90 (SD = 1.53) and for tobacco/nicotine to be 11.26 (SD = 1.67) and 10.91 (SD = 1.99), for males and females, respectively. Although formal statistical analyzes could not be conducted within the current sample given the low rates of cannabis and nicotine use at Time 2, this may suggest that early alcohol sipping is not predictive of cannabis and nicotine use by ages 11, 12. Future research is warranted to examine the association between early alcohol sipping and use of cannabis and nicotine at a later age.

Despite low levels of cannabis and nicotine use at Time 2, 418 youth reported past year alcohol use allowing for examination of the hypothesis that early alcohol sipping is associated with future alcohol use. The hypothesis was supported, and alcohol sipping (in any context) was found to predict future alcohol use, with a medium effect size. This finding is consistent with previous research on alcohol sipping with and without parental permission. Trying sips of alcohol by sixth grade is associated with greater odds of having a full drink, getting drunk, and heavy drinking by ninth grade (17). Additionally, early alcohol sipping with parental approval has been found to predict increased frequency of use and amount of use in late adolescence when controlling for sociocultural and individual differences (59). The primary analyzes included youth who sipped alcohol in any context (e.g., with friends, with parental permission, as part of a religious ceremony) thereby suggesting that any type of alcohol sipping is associated with increased future alcohol use. Additional exploratory analyzes including only youth who sipped outside of religious context revealed similar findings, with alcohol use at Time 1 predicting alcohol use at Time 2, although the overall effect size was small. Overall, these results highlight an association between early alcohol sipping and future alcohol use among youth.

Hypothesis 3 predicted that youth could be classified according to alcohol drinking status at Time 2 based on NA-IFG activation and alcohol sipping at Time 1. This model accurately classified 21% of youth who reported drinking at Time 2. Sipping status at Time 1 was identified as the single most important factor for classification, again highlighting the impact of early alcohol sipping on the trajectory to future alcohol drinking. However, the overall balanced accuracy was only 56% thereby indicating the model was only marginally better than chance at classifying youth. Regardless, these findings contribute to our understanding of alcohol use in youth by highlighting a behavioral relationship between early and future alcohol use while failing to provide support for a neural-behavioral relationship between NA-IFG activation and alcohol sipping. Additionally, this study was the first machine learning examination of adolescent alcohol use to include a measure of sipping. Previous investigations include, Squeglia et al. (41) who used random forest machine learning to examine individual-level precursors of moderate to heavy drinking by age 18 in a sample of substance-naïve youth at ages 12–14. A combination of demographic (e.g., sex, socioeconomic status), behavioral (e.g., early dating), and indicators of brain structure and function were found to be predictive of alcohol use by 18. The present study was limited by the selection of only specific brain regions driven by theory and the ABCD follow-up timeline which only allowed for inclusion of 2-year follow-up data at the time. Given the large range of variables in the ABCD study and the nature of machine learning models, future examinations of early but minimal alcohol use behaviors in the ABCD study should include additional demographic, behavioral, and neuroimaging variables to further clarify the contribution of these various factors to adolescent substance use.

The final hypothesis examined the association of Time 1 NA and IFG activity and alcohol sipping with Time 2 NA and IFG activity; this hypothesis was partially supported. The pattern of results did not suggest the presence of an association between alcohol sipping at a young age and future neural activity in bilateral IFG. However, there was an association between alcohol sipping at ages 9, 10 and NA activity at ages 11, 12; although the effect was very small. There was also a linear relationship between activity at Time 1 and Time 2 in the IFG, while this was not true for NA. This finding may support the imbalance theory which posits that frontal regions of the brain develop in a linear fashion, while NA development is curvilinear, thereby explaining the propensity for risky behaviors in adolescence.

### Implications

Given that trying sips of alcohol at a young age may often be considered inconsequential to many parents and caretakers it is important to understand whether very low-level alcohol use at a young age can be considered an early indicator of vulnerability for future substance use. Although the results of this study do not suggest an association between early alcohol sipping and underlying alternations in brain functioning, they do support an association between early low-level alcohol use and future drinking. Current prevention and intervention efforts highlight the effects of peer pressure and aim to reduce alcohol use among same-aged friends. However, the earliest experiences with alcohol often occur with parental permission as sipping or tasting alcohol may viewed as protective when done in the context of family (59). Adolescents with parental permission to drink alcohol have been found to transition to heavy drinking (5 or more drinks in a sitting) more quickly and to drink heavily more frequently than their peers who do not have parental permission to drink (60). These findings suggest that the parental belief that allowing children to try alcohol with supervision can be protective against future alcohol misuse is inaccurate. Allowing children to drink with parental supervision may actually create an environment where any alcohol use is perceived as permissible and increase frequency use. Ultimately, these findings suggest that early sipping or tasting of alcohol with parental permission is not benign but instead, is a risk for future alcohol misuse. Prevention and intervention efforts should expand to include education for parents about the connection between permitted alcohol experimentation and future misuse.

### Strengths and limitations

A key strength of the current project is the direct examination of youth who report early but minimal sipping. Altered neural activation in the reward-network among adolescents who use substances regularly has consistently been demonstrated (61–63), but this is the first study to examine neural activity among youth who have only tried sips of alcohol. Further, substance use research with youth has been largely cross-sectional, limiting the ability to draw temporal conclusions and make any causal inferences about the role of early use behaviors. The present study attempts to bridge this gap by utilizing data collected as part of the ABCD study to gain a better understanding of the temporal progression of early alcohol sipping, altered neural activity, and future substance use within a large multisite prospective cohort of children in the United States. Furthermore, the use of a robust machine learning technique to identify risk factors for substance use among youth is an additional strength.

Despite the multiple strengths of this project, it is not without its limitations. First, an inherent constraint of task-based fMRI data is the sensitivity to only detect regions engaged by the particular task. While the MID task is widely used and well-validated, other reward tasks may differentially activate NA-IFG in a way more predictive of future substance use. Additionally, other brain regions not associated with reward processing may be more advantageous in the identification of youth at risk for future substance use. Further examinations using machine learning are warranted to clarify if early neural activity is a marker of future substance use. Second, the lack of cannabis and nicotine use reported by youth at Time 2 limited the ability to thoroughly investigate the trajectory of use for these substances. Similarly, although a significant relationship between alcohol use at Time 1 and Time 2 was found, reported alcohol use at Time 2 remains relatively low, making it difficult to fully examine whether early alcohol sipping is associated with progression to more significant alcohol use later (i.e., consuming 1 or more full drinks). Examination of data from future follow-up assessments as some youth increase their substance use will help clarify the predictive value of early but minimal alcohol use.

### Future directions

Although there is a lack of evidence to support the hypothesis that youth self-reported alcohol sipping is a behavioral indicator of any fundamental neural disruption, such a relationship may be obscured by intentional and unintentional underreporting by youth. In a recent analysis of hair toxicology tests from a subsample of at-risk youth who completed the baseline assessment of the ABCD study, 11% had positive toxicology results despite self-reporting no substance use (64). Youth with positive hair toxicology tests for any substance reported underestimated amounts of use while youth with negative results reported more sipping of alcohol than youth with positive tests. Due to apparent youth underreporting of substance use, a link between early alcohol use and neural anomalies can’t be ruled out. Future researchers should continue to examine the relationship between neural dysfunction and early but minimal alcohol use using advanced toxicology screening measures in conjunction with self-report measures.

Other potential avenues for future research include the examination of early alcohol sipping in relation to subsequent neural activation. The present investigation only tested the relationship between alcohol sipping and concurrent reward-related neural activation. Although the results did not demonstrate an association between concurrent alcohol sipping and NA-IFG activation, early alcohol sipping may be associated with neural activity at a later age. The ABCD study is well-poised to examine this question by utilizing data collected at follow-up assessments through the age of 20. Similarly, early alcohol sipping should be examined as a predictor of problematic substance use and substance dependence at future time points. Such research will further elucidate the consequences of early but minimal alcohol use among youth.

### Final conclusion

Overall, the results of this project do not provide strong evidence to suggest that early but minimal use of alcohol is a behavioral marker of underlying alterations in NA-IFG neural responsivity to reward. However, early sips of alcohol, across environmental contexts, appear to predict greater frequency of subsequent alcohol use. Understanding neural and behavioral factors that indicate a greater propensity for future use is crucial for identifying at-risk youth and potential targets for preventative efforts.

## Data availability statement

Publicly available datasets were analyzed in this study. This data can be found here: https://nda.nih.gov/abcd.

## Author contributions

AM, JJ, and ST contributed to manuscript conceptualization. AM and AS completed data cleaning and all statistical analyses. AM prepared manuscript, figures, and tables. JJ, AS, and ST provided revisions and approval of final manuscript. All authors contributed to the article and approved the submitted version.

## References

[1] Johnston LD Miech RA O’Malley PM Bachman JG Schulenberg JE Patrick ME. *Monitoring the Future National Survey Results on Drug use 1975-2018: Overview, key Findings on Adolescent Drug Use.* Ann Arbor, MI: Institute for Social Research (2019).

[2] DeWit DJ Adlaf EM Offord DR Ogborne AC. Age at first alcohol use: a risk factor for the development of alcohol disorders. *Am J Psychiatry.* (2000) 157:745–50. 10.1176/appi.ajp.157.5.745 10784467

[3] Grant BF Dawson DA. Age at onset of alcohol use and its association with DSM-IV alcohol abuse and dependence: results from the national longitudinal alcohol epidemiologic survey. *J Subst Abuse.* (1997) 9:103–10. 10.1016/S0899-3289(97)90009-2 9494942

[4] Hingson RW Heeren T Winter MR. Age at drinking onset and alcohol dependence. *Arch Pediatr Adolesc Med.* (2006) 160:739. 10.1001/archpedi.160.7.739 16818840

[5] Warner LA White HR. Longitudinal effects of age at onset and first drinking situations on problem drinking. *Subst Use Misuse.* (2003) 38:1983–2016. 10.1081/JA-120025123 14677779

[6] Falk DE Yi HY Hilton ME. Age of onset and temporal sequencing of lifetime DSM-IV alcohol use disorders relative to comorbid mood and anxiety disorders. *Drug Alcohol Depend.* (2008) 94:234–45. 10.1016/j.drugalcdep.2007.11.022 18215474 PMC2386955

[7] Hawkins JD Graham JW Maguin E Abbott R Hill KG Catalano RF. Exploring the effects of age of alcohol use initiation and psychosocial risk factors on subsequent alcohol misuse. *J Stud Alcohol.* (1997) 58:280–90. 10.15288/jsa.1997.58.280 9130220 PMC1894758

[8] Shrier LA Emans J Woods ER Durant RH. The association of sexual risk behaviors and problem drug behaviors in high school students. *J Adolesc Health.* (1997) 20:377–83. 10.1016/S1054-139X(96)00180-2 9168385

[9] Rohde P Lewinsohn PM Seeley JR. Psychiatric comorbidity with problematic alcohol use in high school students. *J Am Acad Child Adolesc Psychiatry.* (1996) 35:101–9. 10.1097/00004583-199601000-00018 8567601

[10] Wu P Goodwin RD Fuller C Liu X Comer JS Cohen P The relationship between anxiety disorders and substance use among adolescents in the community: specificity and gender differences. *J Youth Adolesc.* (2010) 39:177–88. 10.1007/s10964-008-9385-5 20084563 PMC2809931

[11] Casey BJ Jones RM. Neurobiology of the adolescent brain and behavior: implications for substance use disorders. *J Am Acad Child Adolesc Psychiatry.* (2010) 49:1189–201. 10.1016/j.jaac.2010.08.017 21093769 PMC3099425

[12] Schweinsburg AD Paulus MP Barlett VC Killeen LA Caldwell LC Pulido C An fMRI study of response inhibition in youths with a family history of alcoholism. *Ann N Y Acad Sci.* (2004) 1021:391–4. 10.1196/annals.1308.050 15251915

[13] Martz ME Cope LM Hardee JE Brislin SJ Weigard A Zucker RA Frontostriatal resting state functional connectivity in resilient and non-resilient adolescents with a family history of alcohol use disorder. *J Child Adolesc Psychopharmacol.* (2019) 29:508–15. 10.1089/cap.2018.0169 31368775 PMC6727473

[14] Hatchard T Mioduszewski O Fall C Byron-Alhassan A Fried P Smith AM. Neural impact of low-level alcohol use on response inhibition: an fMRI investigation in young adults. *Behav Brain Res.* (2017) 329:12–9. 10.1016/j.bbr.2017.04.032 28435127

[15] Maggs JL Staff J Patrick ME Wray-Lake L. Very early drinking: event history models predicting alcohol use initiation from age 4 to 11 years. *Addict Behav.* (2019) 89:121–7. 10.1016/j.addbeh.2018.09.030 30290300

[16] Aiken A Clare PJ Wadolowski M Hutchinson D Najman JM Slade T Age of alcohol initiation and progression to binge drinking in adolescence: a prospective cohort study. *Alcohol Clin Exp Res.* (2018) 42:100–10. 10.1111/acer.13525 29160941

[17] Jackson KM Barnett NP Colby SM Rogers ML. The prospective association between sipping alcohol by the sixth grade and later substance use. *J Stud Alcohol Drugs.* (2015) 76:212–21. 10.15288/jsad.2015.76.212 25785796 PMC5374474

[18] Donovan JE Molina BSG. Childhood risk factors for early-onset drinking*. *J Stud Alcohol Drugs.* (2011) 72:741–51. 10.15288/jsad.2011.72.741 21906502 PMC3174021

[19] Jackson C Ennett ST Dickinson DM Bowling JM. Attributes that differentiate children who sip alcohol from abstinent peers. *J Youth Adolesc.* (2013) 42:1687–95. 10.1007/s10964-012-9870-8 23224982 PMC3622130

[20] Donovan JE Molina BSG. Antecedent predictors of children’s initiation of sipping/tasting alcohol. *Alcohol Clin Exp Res.* (2014) 38:2488–95. 10.1111/acer.12517 25159887 PMC4282024

[21] Geier CF. Adolescent cognitive control and reward processing: implications for risk taking and substance use. *Horm Behav.* (2013) 64:333–42. 10.1016/j.yhbeh.2013.02.008 23998676

[22] Hardin MG Ernst M. Functional brain imaging of development-related risk and vulnerability for substance use in adolescents. *Annu Rev Clin Psychol.* (2016) 11:361–77. 10.1097/ADM.0b013e31819ca788 20161036 PMC2753522

[23] Spear LP. The adolescent brain and age-related behavioral manifestations. *Neurosci Biobehav Rev.* (2000) 24:417–63. 10.1016/S0149-7634(00)00014-2 10817843

[24] Ernst M Torrisi S Balderston N Grillon C Hale EA. fMRI functional connectivity applied to adolescent neurodevelopment. *Annu Rev Clin Psychol.* (2015) 11:361–77. 10.1146/annurev-clinpsy-032814-112753 25581237 PMC4990783

[25] Galvan A Hare TA Parra CE Penn J Voss H Glover G Earlier development of the accumbens relative to orbitofrontal cortex might underlie risk-taking behavior in adolescents. *J Neurosci.* (2006) 26:6885–92. 10.1523/JNEUROSCI.1062-06.2006 16793895 PMC6673830

[26] May J Delgado MR Dahl RE Stenger VA Ryan ND Fiez JA Event-related functional magnetic resonance imaging of reward-related brain circuitry in children and adolescents. *Biol Psychiatry.* (2004) 55:359–66. 10.1016/j.biopsych.2003.11.008 14960288

[27] Van Leijenhorst L Moor BG Op de Macks ZA Rombouts SARB Westenberg PM Crone EA. Adolescent risky decision-making: neurocognitive development of reward and control regions. *NeuroImage.* (2010) 51:345–55. 10.1016/j.neuroimage.2010.02.038 20188198

[28] Van Leijenhorst L Zanolie K Van Meel CS Westenberg PM Rombouts SARB Crone EA. What motivates the adolescent? Brain regions mediating reward sensitivity across adolescence. *Cereb Cortex.* (2010) 20:61–9. 10.1093/cercor/bhp078 19406906

[29] Steinberg L. Risk taking in adolescence: new perspectives from brain and behavioral science. *Curr Direct Psychol Sci.* (2007) 16:55–9. 10.1111/j.1467-8721.2007.00475.x

[30] Steinberg L. A dual systems model of adolescent risk-taking. *Dev Psychobiol.* (2010) 52:216–24. 10.1002/dev.20445 20213754

[31] Delgado MR Nystrom LE Fissell C Noll DC Fiez JA. Tracking the hemodynamic responses to reward and punishment in the striatum. *J Neurophysiol.* (2000) 84:3072–7. 10.1016/0166-2236(90)90107-l 11110834

[32] Elliott R Newman JL Longe OA Deakin JFW. Differential response patterns in the striatum and orbitofrontal cortex to financial reward in humans: a parametric functional magnetic resonance imaging study. *J Neurosci.* (2003) 23:303–7. 10.1523/JNEUROSCI.23-01-00303.2003 12514228 PMC6742125

[33] Ernst M Nelson EE Jazbec S McClure EB Monk CS Leibenluft E Amygdala and nucleus accumbens in responses to receipt and omission of gains in adults and adolescents. *NeuroImage.* (2005) 25:1279–91. 10.1016/j.neuroimage.2004.12.038 15850746

[34] Seymour B Daw N Dayan P Singer T Dolan R. Differential encoding of losses and gains in the human striatum. *J Neurosci.* (2007) 27:4826–31. 10.1523/JNEUROSCI.0400-07.2007 17475790 PMC2630024

[35] May AC Stewart JL Tapert SF Paulus MP. The effect of age on neural processing of pleasant soft touch stimuli. *Front Behav Neurosci.* (2014) 8:52. 10.3389/fnbeh.2014.00052 24600366 PMC3930859

[36] Schneider S Peters J Bromberg S Brassen U Miedl SF Banaschewski T Risk taking and the adolescent reward system: a potential common link to substance abuse. *Am J Psychiatry.* (2012) 169:39–46. 10.1176/appi.ajp.2011.11030489 21955931

[37] Deakin J Aitken MRF Robbins TW Sahakian BJ. Risk taking during decision-making in normal volunteers changes with age. *J Int Neuropsychol Soc.* (2004) 10:590–8. 10.1017/S1355617704104104 15327737

[38] Kuhn M Johnson K. *Applied Predictive Modeling.* New York, NY: Springer (2013). 10.1007/978-1-4614-6849-3

[39] Breiman L. Random forests. *Mach Learn.* (2001) 45:5–32. 10.1023/A:1010933404324

[40] Pisner DA Schnyer DM. Support vector machine. *Mach Learn Methods Appl Brain Disord.* (2020):101–21. 10.1016/B978-0-12-815739-8.00006-7

[41] Squeglia LM Ball TM Jacobus J Brumback T McKenna BS Nguyen-Louie TT Neural predictors of initiating alcohol use during adolescence. *Am J Psychiatry.* (2017) 174:172–85. 10.1176/appi.ajp.2016.15121587 27539487 PMC5288131

[42] Kinreich S Meyers JL Maron-Katz A Kamarajan C Pandey AK Chorlian DB Predicting risk for alcohol use disorder using longitudinal data with multimodal biomarkers and family history: a machine learning study. *Mol Psychiatry.* (2019) 26:1133–41. 10.1038/s41380-019-0534-x 31595034 PMC7138692

[43] Garavan H Bartsch H Conway K Decastro A Goldstein RZ Heeringa S Recruiting the ABCD sample: design considerations and procedures. *Dev Cogn Neurosci.* (2018) 32:16–22. 10.1016/j.dcn.2018.04.004 29703560 PMC6314286

[44] Loeber R Clark DB Ahonen L FitzGerald D Trucco EM Zucker RA. A brief validated screen to identify boys and girls at risk for early marijuana use. *Dev Cogn Neurosci.* (2018) 32:23–9. 10.1016/j.dcn.2018.03.011 29655614 PMC6417103

[45] White N Roddey C Shankaranarayanan A Han E Rettmann D Santos J PROMO: real-time prospective motion correction in MRI using image-based tracking. *Magn Reson Med.* (2010) 63:91–105. 10.1002/mrm.22176 20027635 PMC2892665

[46] Brown TT Kuperman JM Erhart M White NS Roddey JC Shankaranarayanan A Prospective motion correction of high-resolution magnetic resonance imaging data in children. *NeuroImage.* (2010) 53:139–45. 10.1016/j.neuroimage.2010.06.017 20542120 PMC3146240

[47] Kuperman JM Brown TT Ahmadi ME Erhart MJ White NS Roddey JC Prospective motion correction improves diagnostic utility of pediatric MRI scans. *Pediatr Radiol.* (2011) 41:1578–82. 10.1007/s00247-011-2205-1 21779892 PMC3933373

[48] Hagler DJ Hatton S Cornejo MD Makowski C Fair DA Dick AS Image processing and analysis methods for the adolescent brain cognitive development study. *NeuroImage.* (2019) 202:116091. 10.1016/j.neuroimage.2019.116091 31415884 PMC6981278

[49] Sobell LC Sobell MB. Alcohol timeline followback user’s manual. In: ush JA First MB Blacker D editors. *Handbook of Psychiatric Measures.* Washington, DC: American Psychiatric Pub (2000). p. 477–9.

[50] Knutson B Adams CM Fong GW Hommer D. Anticipation of increasing monetary reward selectively recruits nucleus accumbens. *J Neurosci.* (2001) 21:RC159. 10.1523/JNEUROSCI.21-16-j0002.2001 11459880 PMC6763187

[51] Bjork JM Knutson B Fong GW Caggiano DM Bennett SM Hommer DW. Incentive-elicited brain activation in adolescents: similarities and differences from young adults. *J Neurosci.* (2004) 24:1793–802. 10.1523/JNEUROSCI.4862-03.2004 14985419 PMC6730402

[52] Cho YT Fromm S Guyer AE Detloff A Pine DS Fudge JL Nucleus accumbens, thalamus and insula connectivity during incentive anticipation in typical adults and adolescents. *NeuroImage.* (2013) 66:508–21. 10.1016/j.neuroimage.2012.10.013 23069809 PMC3949208

[53] Casey BJ Cannonier T Conley MI Cohen AO Barch DM Heitzeg MM The adolescent brain cognitive development (ABCD) study: imaging acquisition across 21 sites. *Dev Cogn Neurosci.* (2018) 32:43–54. 10.1016/j.dcn.2018.03.001 29567376 PMC5999559

[54] Desikan RS Ségonne F Fischl B Quinn BT Dickerson BC Blacker D An automated labeling system for subdividing the human cerebral cortex on MRI scans into gyral based regions of interest. *NeuroImage.* (2006) 31:968–80. 10.1016/j.neuroimage.2006.01.021 16530430

[55] Fischl B Salat DH Busa E Albert M Dieterich M Haselgrove C Whole brain segmentation: automated labeling of neuroanatomical structures in the human brain. *Neuron.* (2002) 33:341–55. 10.1016/S0896-6273(02)00569-X 11832223

[56] Braams BR Peper JS Van Der Heide D Peters S Crone EA. Nucleus accumbens response to rewards and testosterone levels are related to alcohol use in adolescents and young adults. *Dev Cogn Neurosci.* (2016) 17:83–93. 10.1016/j.dcn.2015.12.014 26771250 PMC4722250

[57] Rioux C Castellanos-Ryan N Parent S Vitaro F Tremblay RE Séguin JR. Age of cannabis use onset and adult drug abuse symptoms: a prospective study of common risk factors and indirect effects. *Can J Psychiatry.* (2018) 63:457–64. 10.1177/0706743718760289 29682999 PMC6099774

[58] Malmberg M Overbeek G Monshouwer K Lammers J Vollebergh WAM Engels RCME. Substance use risk profiles and associations with early substance use in adolescence. *J Behav Med.* (2010) 33:474–85. 10.1007/s10865-010-9278-4 20625809 PMC2967704

[59] Colder CR Shyhalla K Frndak SE. Early alcohol use with parental permission: psychosocial characteristics and drinking in late adolescence. *Addict Behav.* (2018) 76:82–7. 10.1016/j.addbeh.2017.07.030 28772246 PMC5614833

[60] Staff J Maggs JL. Parents allowing drinking is associated with adolescents’ heavy alcohol use. *Alcohol Clin Exp Res.* (2020) 44:188–95. 10.1111/acer.14224 31750959 PMC6980970

[61] Brumback T Squeglia LM Jacobus J Pulido C Tapert SF Brown SA. Adolescent heavy drinkers’ amplified brain responses to alcohol cues decrease over one month of abstinence. *Addict Behav.* (2015) 46:45–52. 10.1016/j.addbeh.2015.03.001 25796007 PMC4395544

[62] Heitzeg MM Cope LM Martz ME Hardee JE. Neuroimaging risk markers for substance abuse: recent findings on inhibitory control and reward system functioning. *Curr Addict Rep.* (2015) 2:91–103. 10.1007/s40429-015-0048-9 26236575 PMC4520315

[63] Tapert SF Cheung EH Brown GG Frank LR Paulus MP Schweinsburg AD Neural response to alcohol stimuli in adolescents with alcohol use disorder. *Arch Gen Psychiatry.* (2003) 60:727. 10.1001/archpsyc.60.7.727 12860777

[64] Wade NE Tapert SF Lisdahl K Huestis M Haist F. Hair toxicology identifies greater substance use than self-report in high-risk youth enrolled in the ABCD Study. *Proceedings of the to be Presented at the Annual Conference of the College on Problems of Drug Dependence.* Scottsdale, AZ: (2021).

[65] Barch DM Albaugh MD Avenevoli S Chang L Clark DB Glantz MD Demographic, physical and mental health assessments in the adolescent brain and cognitive development study: rationale and description. *Dev Cogn Neurosci.* (2018) 32:55–66. 10.1016/j.dcn.2017.10.010 29113758 PMC5934320

[66] Knutson B Westdorp A Kaiser E Hommer D. FMRI visualization of brain activity during a monetary incentive delay task. *NeuroImage.* (2000) 12:20–7. 10.1006/nimg.2000.0593 10875899

[67] Yau W-YW Zubieta J-K Weiland BJ Samudra PG Zucker RA Heitzeg MM. Nucleus accumbens response to incentive stimuli anticipation in children of alcoholics: relationships with precursive behavioral risk and lifetime alcohol use. *J Neurosci.* (2012) 32:2544–51. 10.1523/JNEUROSCI.1390-11.2012 22396427 PMC3567451

